# Identification of Interface Structure for a Topological CoS_2_ Single Crystal in Oxygen Evolution Reaction with High Intrinsic Reactivity

**DOI:** 10.1021/acsami.1c24966

**Published:** 2022-04-25

**Authors:** Yu Kang, Yangkun He, Darius Pohl, Bernd Rellinghaus, Dong Chen, Marcus Schmidt, Vicky Süß, Qingge Mu, Fan Li, Qun Yang, Hedong Chen, Yufei Ma, Gudrun Auffermann, Guowei Li, Claudia Felser

**Affiliations:** † Max Planck Institute for Chemical Physics of Solids, Nöthnitzer Str. 40, 01187 Dresden, Germany; ‡ Dresden Center for Nanoanalysis, cfaed, Technische Universität Dresden, Helmholtzstraße 18, 01069 Dresden, Germany; § Max Planck Institute for Microstructure Physics, Weinberg 2, D-06120 Halle, Sachsen-Anhalt, Germany; ∥ CAS Key Laboratory of Magnetic Materials and Devices, and Zhejiang Province Key Laboratory of Magnetic Materials and Application Technology, Ningbo Institute of Materials Technology and Engineering, Chinese Academy of Sciences, Ningbo 315201, China; ⊥ University of Chinese Academy of Sciences, Shijingshan District, Beijing 100049, China

**Keywords:** cobalt disulfide, topological metal, oxygen evolution reaction, interface structure, cobalt oxide

## Abstract

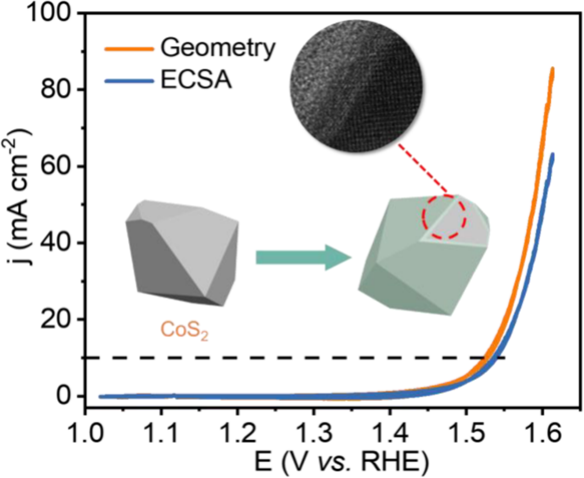

Transition metal chalcogenides such as CoS_2_ have been reported as competitive catalysts for oxygen evolution reaction. It has been well confirmed that surface modification is inevitable in such a process, with the formation of different re-constructed oxide layers. However, which oxide species should be responsible for the optimized catalytic efficiencies and the detailed interface structure between the modified layer and precatalyst remain controversial. Here, a topological CoS_2_ single crystal with a well-defined exposed surface is used as a model catalyst, which makes the direct investigation of the interface structure possible. Cross-sectional transmission electron microscopy of the sample reveals the formation of a 2 nm thickness Co_3_O_4_ layer that grows epitaxially on the CoS_2_ surface. Thick CoO pieces are also observed and are loosely attached to the bulk crystal. The compact Co_3_O_4_ interface structure can result in the fast electron transfer from adsorbed O species to the bulk crystal compared with CoO pieces as evidenced by the electrochemical impedance measurements. This leads to the competitive apparent and intrinsic reactivity of the crystal despite the low surface geometric area. These findings are helpful for the understanding of catalytic origins of transition metal chalcogenides and the designing of high-performance catalysts with interface-phase engineering.

## 1 Introduction

Energy consumption together with CO_2_ emission has caused global concerns over the decades, this has given rise to the targets of peak carbon emission and carbon neutrality recently.^1−3^ Electrochemical water splitting is one of the best possible solutions that transfer renewable energy into green hydrogen fuel.^4−6^ The oxygen evolution reaction (OER) in water splitting is the rate-determining step because it is a complex four-electron process with high overpotential.^7−10^ Many advanced nanocatalysts have been developed to promote the performance of OER, such as transition metal oxides, hydroxides, chalcogenides, and phosphides.^11−16^ Among them, transition metal sulfides, usually with extremely low cost and high electric conductivity, are highly potential catalysts.^17−19^ These nanosulfides may experience surface reconstruction at high potential during OER and are recognized as precatalysts.^20,21^ In this case, the electron derived from adsorption oxygen species would transfer through surface-reconstructed layers and inner bulk precatalyst before reaching the electrode substrate. Therefore, both the reconstructed layer and bulk precatalysts play a role in promoting the performance, forming a so-called active structure.^22−24^ However, previous works usually focused on identifying either the bulk sulfides or the reconstructed species. Little is known about the interface between the surface reconstructed layer and bulk precatalysts. This may be due to the complex surface structure of nanocatalysts, giving rise to the difficulty in understanding the interface. Hence, well-defined sulfide electrocatalysts with good performance are highly desired.

Recently, booming topological materials, which are also single crystals, seem to provide a new approach. Topological single crystals such as PtSn_4_, 1T′-MoTe_2_, Co_3_Sn_2_S_2_, and so forth have been introduced into water splitting.^25−29^ These semimetals have extremely high charge carrier mobility and a protected topological surface state derived from the bulk band inversion of the valence and conduction band.^30−32^ Such special properties can facilitate the electron transfer between the bulk crystal and adsorbates on the surface.^33−35^ Recently, it was reported that the CoS_2_ single crystal hosts a topological nodal line and Fermi arc surface state in the band structure close to the Fermi level (*E*_f_); therefore, it is also verified as a topological material.^36^ This suggests it might be a good electrocatalyst with superior electric conductivity. As far as we know, the CoS_2_ single crystal has not yet been studied for water splitting, making it an ideal model catalyst to clarify the interface structure for OER.

In this work, we have successfully grown a well-crystallized topological CoS_2_ single crystal, which exhibits superior intrinsic reactivity based on its effective electrochemical surface area. Assisted by the focused ion beam (FIB) technique, electron microscopy, and electrochemical analysis, the surface evolution species, that is, CoO pieces and Co_3_O_4_, have been clearly identified after OER. The Co_3_O_4_ is observed to epitaxially grow on the bulk surface, such that the Co_3_O_4_–CoS_2_ with a compact interface could facilitate fast electron transfer and promote the intrinsic performance. Therefore, the CoS_2_ single crystal acts as a clean model catalyst for studying surface evolution and the interface structure, paving a way for further understanding of the electron transfer process between adsorbed O species and catalysts for OER.

## 2 Experimental Section

### 2.1 Synthesis of a CoS_2_ Single Crystal

Single crystals of CoS_2_ were grown *via* chemical transport reaction using CoBr_2_ as the transport additive. CoS_2_ had first been synthesized by a direct reaction of elemental cobalt (powder 99.998%, Alfa Aesar) and sulfur (pieces 99.99%, Alfa Aesar) at 700 °C in an evacuated fused silica tube for 7 days. Starting from this microcrystalline powder, CoS_2_ was crystallized by a chemical transport reaction in a temperature gradient from 700 °C (source) to 640 °C (sink), with the addition of 3.5 mg/mL tube volume cobalt(II) bromide (Alfa Aesar 99.99%, ultradry). After 14 days, the experiment was stopped by quenching the ampoule in cold water. The obtained crystals showed a well-developed morphology. The preparation of RuO_2_, Co_3_O_4,_ and CoOOH is shown in the Supporting Information.

### 2.2 Characterizations

CoS_2_ single crystal formation was confirmed with a pyrite structure by powder X-ray diffraction (XRD) using a monochromator’s Co Kα radiation and the Laue diffraction method. The surface morphology of the crystal was characterized by scanning electron microscopy (SEM) (Philips XL30). High resolution transmission electron microscopy (HRTEM) and energy-dispersive X-ray analysis were carried out by using a JEOL F200 with an operating voltage of 200 kV. Electron transparent samples were prepared by the FIB technique using an FEI Helios 660. Resistivity measurement was conducted on quantum design PPMS *via* the four-probe method. X-ray photoelectron spectroscopy (XPS) spectra were obtained from a UHV surface analysis system equipped with a Scienta-200 hemispherical analyzer. Raman spectra studies were carried out by a customary confocal micro-Raman spectrometer with an unpolarized HeNe laser (632.8 nm) as the light source.

### 2.3 Electrochemical Measurement

The electrochemical measurement was carried out on an Autolab PGSTAT302N electrochemistry workstation. Three electrode system was employed. A carbon rod and a Ag/AgCl electrode were used as the counter electrode and reference electrode, respectively, with 1 M KOH solution as the electrolyte. The solution was bubbled before and during the test. For the working electrode preparation, the CoS_2_ single crystal was attached with Ti wire by a silver paint, which was covered by resin after it dried up. Linear sweep voltammetry (LSV) was measured at a scan rate of 5 mV/s to avoid the interference of the capacitive current. The electrochemical impedance spectroscopy was conducted from 10 kHz to 0.01 Hz. The applied potential against the reference electrode was converted into potential *versus* RHE by
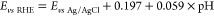
1

The effective electrochemical active surface area (ECSA) was calculated by
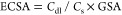
2where the double-layer capacitance (*C*_dl_) was obtained by plotting the difference of current density Δ*J* = (*J*_anodic_ – *J*_cathodic_)/2 at 1.17 V *versus* RHE against the scan rate. *C*_s_ is the specific capacitance, which is estimated to be 0.02 mF/cm^2^,^37^ and GSA is the geometric surface area of the single crystal.

## 3 Results and Discussion

### 3.1 Crystal Structure

**Figure 1:**
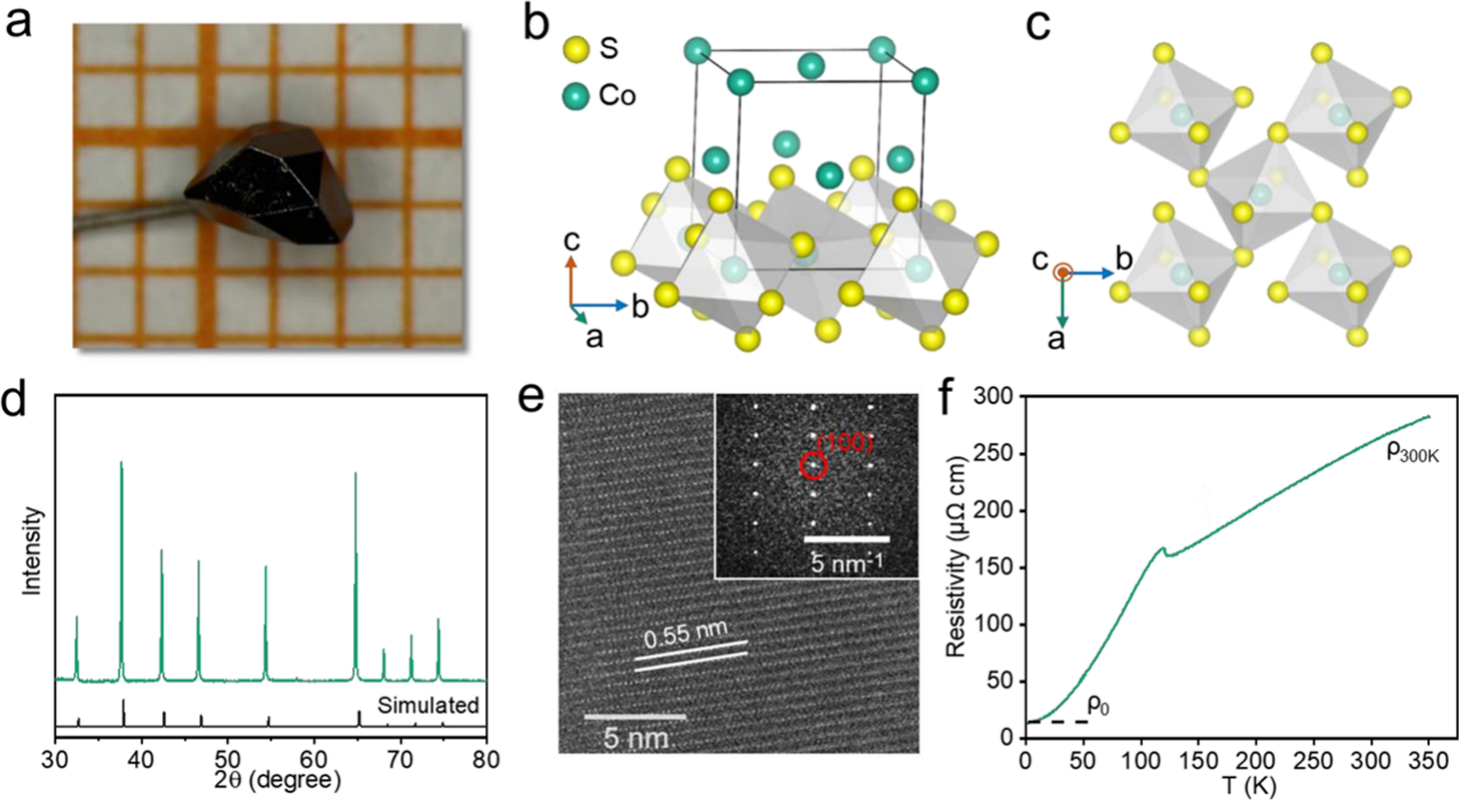
(a)Photograph and (b,c) pyrite structure of the CoS_2_ single crystal. (d) XRD pattern, (e) HRTEM with the corresponding FFT image, and (f) resistivity *vs* temperature curve of the CoS_2_ crystal.

We have successfully prepared the CoS_2_ single crystal by the chemical vapor transport (CVT) method (see Experimental Section). The obtained well-crystallized polyhedron was around 2 mm in size (Figure 1a). The Laue diffraction pattern presenting clear ordered diffraction spots confirmed the high quality of the single crystal (Figure S1). CoS_2_ exists in the pyrite cubic structure (Figure 1b), with corner-shared [CoS_6_] connecting it together (Figure 1c), which could be expected as a metallic state owing to the octahedral Co^2+^ sites with 3d^7^ configuration. The powder XRD pattern has demonstrated the CoS_2_ phase with the space group of *Pa*3̅ (no. 205) (Figure 1d). The crystal structure was further confirmed by HRTEM and the corresponding fast Fourier transform (FFT) (Figure 1e), which presented clear lattice fringes of 0.55 nm distance, corresponding to the (100) plane of CoS_2_. In addition, the resistivity *versus T* curve of CoS_2_ is given in Figure 1f. The crystal exhibited typical metallic property owing to the increasing resistivity with temperature, although there was a resistivity slope variation at 120 K, ascribed to the phase transition from ferromagnetic to the paramagnetic state. The crystal presented around 260 μΩ·cm at room temperature, indicating excellent electric conductivity, which can facilitate electron transfer during electrochemistry. The residual resistivity ratio, defined as ρ_300K_/ρ_0_, was around 17.6, also suggesting the high crystallinity of CoS_2_.

### 3.2 OER Performance and Characterizations

**Figure 2:**
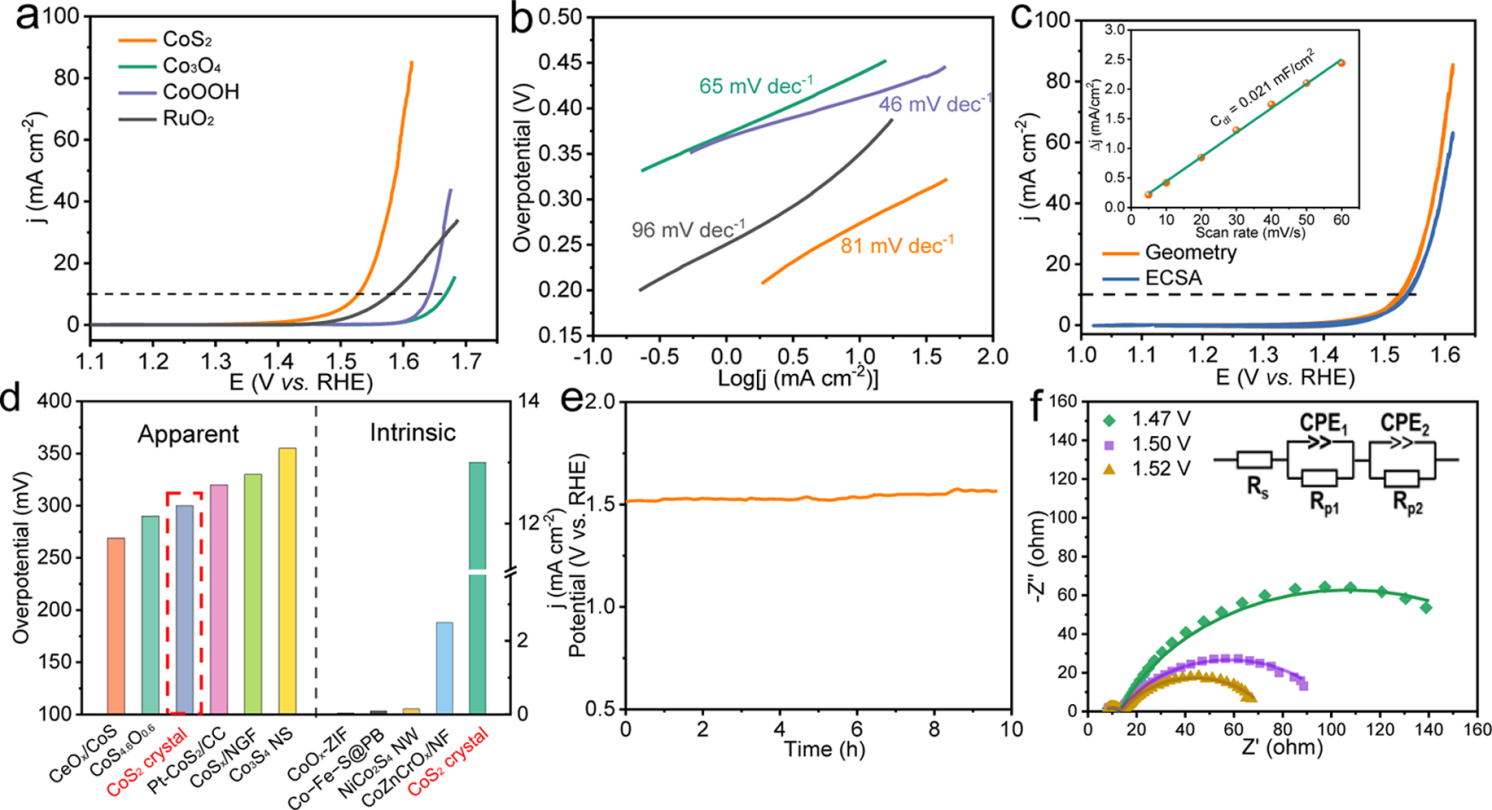
OER performance of the CoS_2_ single crystal and reference materials with 90% *iR* correction. (a) LSV curves with the scanning rate of 5 mV s^–1^ and (b) Tafel slope (CoS_2_ was tested at the steady state from 1.40 to 1.66 V at the interval of 0.02 V). (c) Cyclic voltammetry results comparison based on the geometric area and ECSA of the single crystal. The inset is the difference in current density between anodic and cathodic sweeps *vs* scan rate. (d) Apparent overpotential comparison (left) at 10 mA cm^–2^ normalized to the geometric surface area and the intrinsic current density comparison (right) at 1.55 V based on ECSA. (e) Stability test at a static current density of 10 mA cm^–2^. (f) Nyquist plots under different potentials. The inset shows the equivalent circuit for the two semicircles.

The OER performance of the CoS_2_ single crystal was studied in 1 M KOH electrolyte, and the results are shown in Figure 2. Typical RuO_2_, Co_3_O_4_, and CoOOH powders were also compared by using a rotating disk electrode in the same solution. Linear sweep voltammetry (LSV) presented that the overpotential for CoS_2_ at 10 mA cm^–2^ was around 300 mV (Figure 2a), much lower than those reference RuO_2_, Co_3_O_4_, and CoOOH materials alone. This indicated that CoS_2_ can be a good precatalyst for OER, although the Tafel slope of the CoS_2_ crystal was 81 mV dec^–1^ (Figure 2b), slightly higher than the value of typical CoOOH and Co_3_O_4_ (46 and 65 mV dec^–1^) in water splitting. To reveal the intrinsic OER performance, we measured the effective ECSAs of the single crystal, as shown in Figure S2, and the electrochemical double-layered capacitance (*C*_dl_) was estimated to be only 0.021 mF cm^–2^ (inset in Figure 2c), almost orders of magnitude lower than those of other nanomaterials reported. It is interesting to note that such a low *C*_dl_ has still ensured a superior activity of CoS_2_. The specific cyclic voltammetry normalized to ECSA showed that the overpotential only increased slightly to 310 mV, similar to the geometric surface-normalized results, indicating that the ECSA was almost approximate to the surface area. We compared the results with previously reported nanomaterials; as shown in Figure 2d, the single crystal exhibited competitive apparent overpotential, and most importantly, the current density of CoS_2_ based on ECSA was nearly orders of magnitude higher than other works under the potential of 1.55 V, demonstrating its excellent intrinsic electrochemical activity for OER. The stability was also investigated at a static current density of 10 mA cm^–2^ (Figure 2e). A relatively stable performance can be guaranteed, with no obvious potential increase during 10 h. The faradaic efficiency of CoS_2_ was around 90% (Figure S3), indicating the slow oxidation of the crystal during OER, which may affect the long-term stability of CoS_2_.

Electrochemical impedance spectroscopy (EIS) was used to investigate the electron transfer process. The measurement was carried out at different potentials in the OER region. Two obvious semicircles in the Nyquist plots in Figure 2f can be observed and fitted with two time constants in series with each other. The semicircle at low frequency was effectively influenced by the applied overpotential, with a smaller *R*_p2_ value at higher potential (Table S1), which can be ascribed to the charge transfer process of OER between the catalyst and adsorption species. Additionally, the small semicircle at high frequency was almost independent concerning the applied potential due to the small change in *R*_s_ (also see Figure S4 and Table S1). This might be related to the fast electron transfer from the bulk single crystal to the surface active layers, in which the high electrical conductivity of CoS_2_ played an important role.^38^

**Figure 3:**
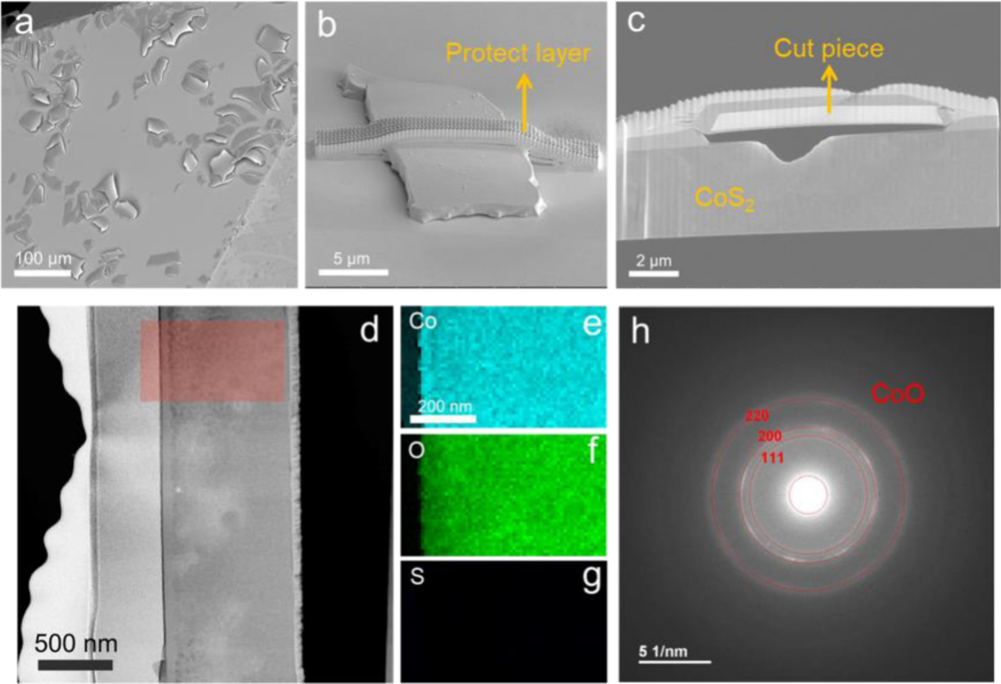
SEM image of (a) surface pieces on the CoS_2_ single crystal after OER, (b) one of the pieces after depositing the protective layer for FIB, and (c) cross section of the cut piece after FIB. (d) STEM annular dark-field (ADF) image of the cross-section and (e–g) corresponding EDS elemental mappings. (h) SAED pattern of the cut piece, which was fitted with (111), (200), and (220) reflections of CoO.

Compared with nano-electrocatalysts, the single crystal can provide a better platform to understand the mechanism of high intrinsic reactivity of CoS_2_ and its real active structure during water splitting. The SEM image in Figure 3a showed that there were many sheet-like pieces with the size of around 50 μm stacked on the crystal surface after OER. This might be the oxidation product. We used the FIB technique to cut the piece and expose the cross section (see details in Figure S5). Before cutting, the protective layer was first deposited onto the piece (Figure 3b). It can be seen from the cross section that there was a gap between the surface piece and bulk CoS_2_ (Figure 3c), and this is not facile to charge transfer between them. Figure 3d–g presents the scanning TEM (STEM) images and the corresponding energy-dispersive X-ray spectroscopy (EDS). In the red rectangle area of the cross section, the sulfur element mapping has disappeared, with only Co and O left in the cut piece, demonstrating that the crystal was indeed oxidized forming pieces of the layer on the surface during water splitting. We performed the selected area electron diffraction (SAED) measurements and had a fit (Figure 3h). As a result, the cut piece could be attributed to the textured nanocrystalline CoO (ICSD #9865) owing to the appearance of broad (200) reflections and otherwise ring patterns.

**Figure 4:**
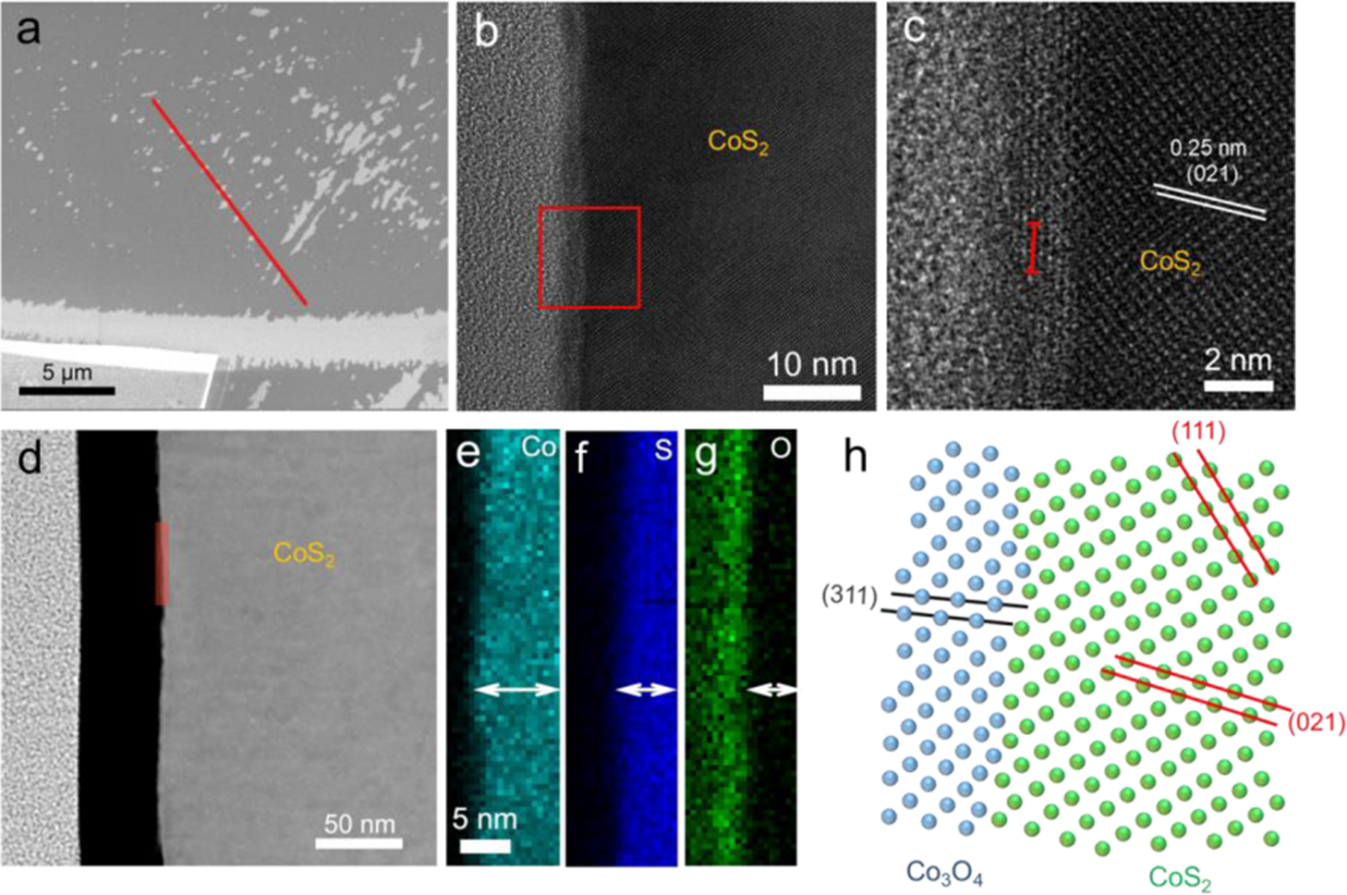
SEM image of (a) thin oxidation layer on CoS_2_ surface after OER. (b) High-resolution TEM of the cross section of the surface oxidation layer (c) and the corresponding amplified area in the red square frame. (d) STEM ADF of the cross section and (e–g) corresponding EDS elemental mappings of the marked area in (d). (h) Illustration of the interface of Co_3_O_4_ and CoS_2_ crystal in the TEM.

Besides the CoO on the single crystal, there is still a question considering the state of other surface places without pieces. Therefore, we cut the surface of CoS_2_ after OER along the red line, as shown in Figure 4a, by the FIB technique (see details in Figure S6). Figure 4b shows the TEM of the cut cross section, with an obvious shell layer covered on the surface of CoS_2_. Figure 4c clearly presents the lattice stripes of the shell. It was observed that the shell closely grew on the surface of the bulk crystal, which was different from those pieces of CoO. We measured the lattice distance along the red line, and the profile is given in Figure S7, which gave a plane distance of around 0.24 nm, consistent with the (311) plane of the Co_3_O_4_ phase.^39^ STEM and EDS mappings of near surface in Figure 4d–g showed that the Co element layer was thicker than the S layer, and the O layer had indeed overlapped with the Co layer. This further demonstrated the formation of the surface oxidation layer. Line scanning also indicated the existence of a surface layer (Figure S8). Therefore, it can be concluded that the Co_3_O_4_ layer formed on the surface after OER. In addition, we also found that the distance in the (311) plane of cubic Co_3_O_4_ (0.24 nm) along the red line direction was approximately equal to the (021) plane spacing of cubic CoS_2_ (Figure 4c), although the lattice of Co_3_O_4_ was not much clear, suggesting that the Co_3_O_4_ layer seemed to epitaxially grow on the bulk crystal, as illustrated in Figure 4h. This kind of structure may promote electron transfer and contribute to the superior intrinsic reactivity of the CoS_2_ crystal.

**Figure 5:**
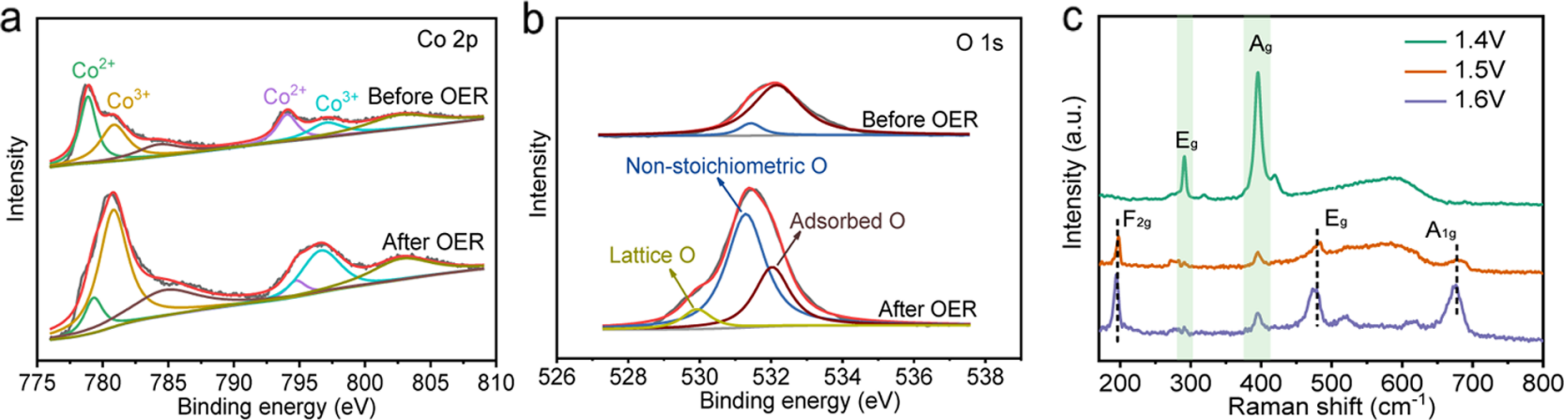
XPS spectra of (a) Co 2p and (b) O 1s. (c) Raman spectra of the CoS_2_ crystal after the measurements at 1.4, 1.5, and 1.6 V.

We compared the XPS of the samples before and after OER with the XPS survey, as shown in Figure S9. The Co spectrum of the fresh one (Figure 5a) was deconvoluted, and the main binding energy at 778.9 and 794.1 eV were ascribed to 2p_3/2_ and 2p_1/2_ of Co^2+^ in CoS_2_ single crystal, respectively.^40^ The smaller peaks at 780.8 and 797.0 eV can be attributed to the Co^3+^ species due to the slight oxidation of the surface. The left two peaks at 784.6 and 802.9 eV correspond to the Co 2p satellites. After OER, it can be observed that the whole Co 2p_3/2_ peak shifted into higher binding energy, consistent with previous results.^24^ The resulting area of Co^3+^ species became much larger than that of Co^2+^, further demonstrating the existence of the oxidation layers. In addition, the O 1s spectrum of fresh and reacted crystal was also deconvoluted into two peaks and three peaks, respectively (Figure 5b). The binding energy at 532.1 eV was assigned to the adsorbed water species,^41^ with similar peak areas for the two samples. The binding energy at 531.4 eV was associated with the nonstoichiometric oxygen on the surface. Here, for the reacted sample, both CoO and Co_3_O_4_ might contribute to this peak due to their defects, leading to a much larger area than that of the fresh one. For the peak at 529.9 eV after OER, it was produced from the lattice oxygen of the surface cobalt oxides pieces. Hence, the XPS analysis was consistent with the results of electron microscopy. Besides, we compared the O 1s spectra of this work and those of the pure CoOOH.^42^ It was found that their peak shapes were rather different from each other, indicating the O species did not come from CoOOH. On the contrary, the O 1s peak shape was much similar to the result of the Co_3_O_4_ structure (Figure S10).^43^ This also suggested the existence of Co_3_O_4_.

To further understand the oxidation of the single crystal at different potentials, a Raman spectrum was further carried out. As shown in Figure 5c, there were two main peaks after the potentiostatic measurement at 1.4 eV, which can be assigned to the A_g_ and E_g_ vibration modes of the CoS_2_ structure,^44^ indicating that very little oxidation has occurred on the surface. When increasing the potential to 1.5 and 1.6 V, besides the CoS_2_ phase, another three vibration peaks ascribing to the F_2g_, E_g_, and A_1g_ Raman active modes of Co_3_O_4_ were also observed.^45^ This suggested that the surface oxidation layer quickly generated at above 1.5 V. Besides, the significantly increased OER performance over 1.5 V indicated that the Co_3_O_4_ layer formation may play an important role in water splitting.

### 3.3 Discussion

Although previous work reported the surface evolution of nanocatalysts during water splitting, there is still a lack of knowledge about the detailed interface structure between the surface reconstructed layer and the bulk precatalysts due to their complex structure. In this work, we used the topological CoS_2_ single crystal with excellent conductivity as the model catalyst and identified two kinds of oxidation species on the surface of CoS_2_ after OER. They are the CoO pieces and Co_3_O_4_. Most importantly, it was revealed that the thin Co_3_O_4_ layer epitaxially grew on the CoS_2_ surface (Figure 4b,c), while the pieces of CoO were loosely attached to the crystal (Figure 3a). Thus, such a compact Co_3_O_4_–CoS_2_ interface would be more facile for the transfer of electrons between single crystal and adsorbed O species, which has already been demonstrated by the very small semicircle at high frequency in Figure S4. In addition, the intrinsic activity based on ECSA was quite close to the geometric surface normalized results (Figure 2c), indicating that the ECSA is approximate to the geometric area. From the abovementioned discussion, we know the Co_3_O_4_ thin layer is covered on the CoS_2_ geometric surface. This further demonstrated that Co_3_O_4_–CoS_2_ with a compact interface formed the active structure for OER instead of the CoO pieces. Although some work has indicated that Co_3_O_4_ can be transformed into CoOOH during the reaction,^46−49^ only our result has definitively identified Co_3_O_4_ species on the surface after OER. This might be due to the reversible transformation between the oxy-hydroxide and Co_3_O_4_ layer (Co_3_O_4_ + OH^–^ + H_2_O ↔ 3CoOOH + e^–^) at a different potential,^50,51^ and the compact interface between Co_3_O_4_ and bulk crystal could facilitate such a fast transformation and thus contribute to the high performance. Therefore, we can conclude that the Co_3_O_4_–CoS_2_ with such a compact interface was the active structure owing to the fast and efficient electron transfer during OER.

## 4 Conclusions

In summary, we have successfully grown high-quality topological CoS_2_ single crystal *via* the CVT method, which exhibited superior intrinsic OER performance compared with nanocounterparts. Further electron microscopes with the FIB technique identified two kinds of oxidation species in situ formed on the surface after the reaction, that is, the CoO pieces and Co_3_O_4_. The Co_3_O_4_ thin layer with several nanometers grew epitaxially on the surface of the conductive CoS_2_ crystal, facilitating the electron transfer between bulk crystal and adsorbed O species. Thus, the formed Co_3_O_4_–CoS_2_ with a compact interface was revealed as the active structure for OER and contributed to the high intrinsic reactivity. Overall, this work reveals the interface structure of CoS_2_ for OER and helps in further understanding of the connection between surface evolution species and bulk precatalysts for efficient electron transfer.

## Supporting Information Available

Additional experimental details, Laue diffraction, electrochemical OER results, electron microscopy, XPS, and the fitting parameters of EIS (PDF)
