# Supplementary material for: Identification of Interface Structure for a Topological CoS2 Single Crystal in Oxygen Evolution Reaction with High Intrinsic Reactivity
Source: ACS Appl Mater Interfaces. 2022 Apr 25;14(17):19324–31. doi: 10.1021/acsami.1c24966 (PMC9073842; doi:10.1021/acsami.1c24966)
Supplement: Supplementary file 1 — am1c24966_si_001.pdf [file am1c24966_si_001.pdf]

Supporting information

Identification of interface structure for topological CoS_2 single crystal in oxygen evolution reaction with high intrinsic reactivity

Yu Kang [a,*], *Yangkun He* [a], *Darius Pohl* [b], *Bernd Rellinghaus* [b], *Dong Chen* [a], *Marcus Schmidt* [a], *Vicky Süß* [a], *Qingge Mu* [a], *Fan Li* [c], *Qun Yang* [a], *Hedong Chen* [a], *Yufei Ma* [a], *Gudrun Auffermann* [a], *Guowei Li* [d,e,*], *Claudia Felser* [a,*]

[a] Max Planck Institute for Chemical Physics of Solids, Nöthnitzer Str. 40, 01187 Dresden, Germany.

[b] Dresden Center for Nanoanalysis, cfaed, Technische Universität Dresden, Helmholtzstraße 18, 01069 Dresden, Germany.

[c] Max Planck Institute for Microstructure Physics, Weinberg 2, D-06120 Halle, Sachsen-Anhalt, Germany.

[d] CAS Key Laboratory of Magnetic Materials and Devices, and Zhejiang Province Key Laboratory of Magnetic Materials and Application Technology, Ningbo Institute of Materials Technology and Engineering, Chinese Academy of Sciences, Ningbo 315201, China.

[e] University of Chinese Academy of Sciences, Shijingshan District, Beijing 100049, China.
Corresponding authors: Yu.Kang@cpfs.mpg.de; liguowei@nimte.ac.cn; Claudia.Felser@cpfs.mpg.de

The supporting information contains additional experimental details, structure (Fig. S1), electrochemical OER results (Fig. S2-4), electron microscopy (Fig. S5-8), XPS (Fig. S9, 10), the fitting parameters of EIS (Table S1) and references.

1. Additional experimental details

1.1 Reference materials preparation

In a typical synthesis of CoOOH, 50 mL of 0.1 M NaOH was added dropwise into 80 mL of 0.05 M $Co(NO_3)_2$ at 45 °C of water bath. The precipitate was filtered and washed for several times, and then dispersed in 40 mL water at 45 °C. 5 mL of 8 M NaOH and 4 mL of 30% H_2O_2 mixture were added dropwise under stirring. After keeping at 45 °C for 18 h, the precipitate was filtered and washed with deionized water, then dried at 65 °C for around 2 days. The final product was CoOOH.

For synthesis of RuO_2, a sol-gel method was used. 2 mmol $RuCl_3 \cdot xH_2O$ and 6 mmol citric acid were mixed in 30 mL water, followed by adding 9 mmol ethylene glycol. The solution was stirred for 1 h, and then put into 80 °C water bath under stirring until the gel formation. The gel was dried at 120 °C for 2 days, finally calcined at 600 °C for 6 h, collected as RuO_2.

The Co_3O_4 powder was purchased from Sigma-Aldrich.

1.2 Working electrode preparation

For RuO_2 or Co_3O_4 electrode, 5 mg of the material and 1 mg of Vulcan XC 72 carbon black were dispersed in 460 µl C_2H_5OH and 500 µl water solution, followed by adding 40 µl of 5% Nafion solution to form homogeneous mixture under sonication. 6 µL of the mixture was drop-casted onto a rotating disk electrode (RDE) with a diameter of 3 mm. For CoOOH electrode, 5 mg of CoOOH and 2 mg of Vulcan XC 72 carbon black were dispersed in 960 µl C_2H_5OH, 1000 µl water, and 40 µl of 5% Nafion solution. 12 µL of the mixture was drop-casted onto a RDE. The resulting catalyst loading of RuO_2, Co_3O_4, and CoOOH was ~0.42 mg/cm^2.

2. Additional Figures

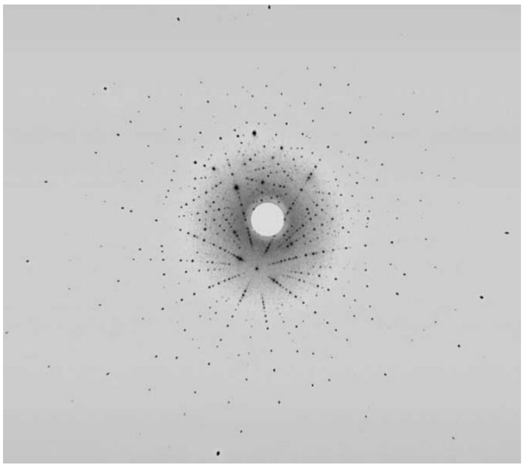

Figure S1. Laue diffraction pattern of CoS_2 single crystal.

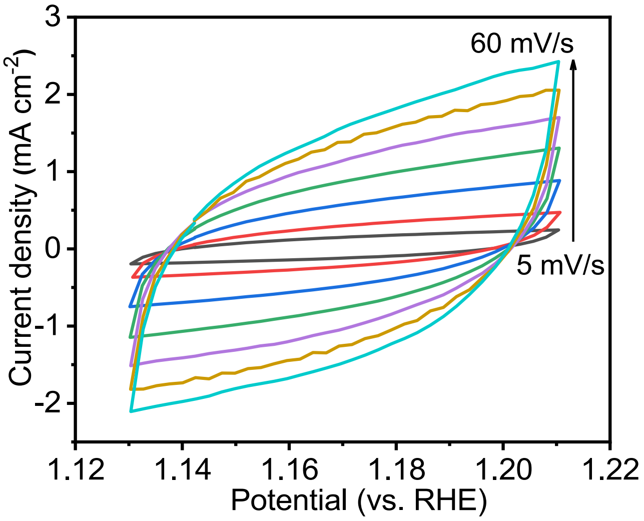

Figure S2. Cyclic voltammetry curves measured at the non-Faradaic region with different scan rate.

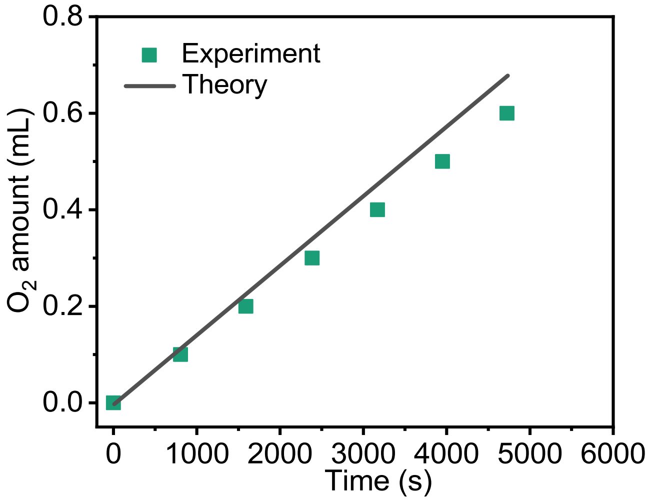

Figure S3. Theoretical and experimental amount of oxygen produced at the static current of 2.5 mA.

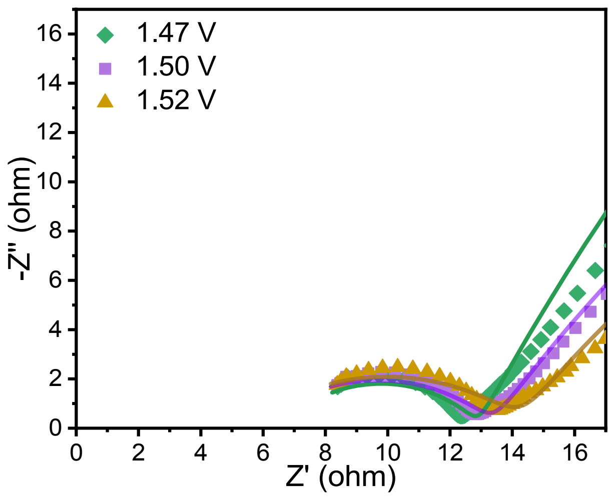

Figure S4. Enlarged Nyquist plot at high frequency.

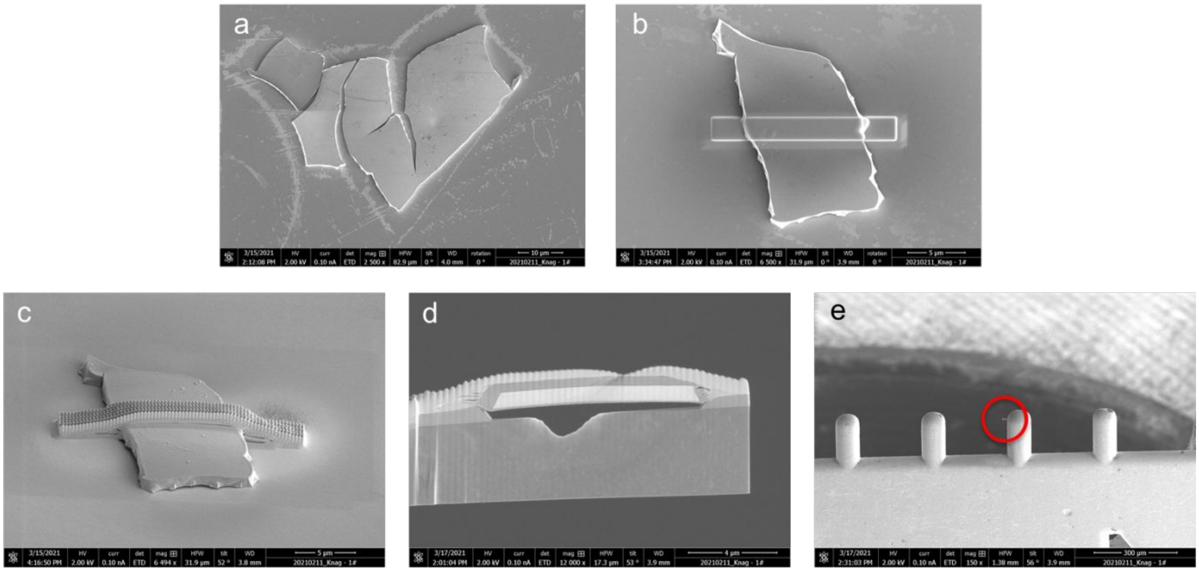

Figure S5. The focused ion beam (FIB) process to obtain the lamella by cutting the surface pieces and exposing the cross section for TEM.

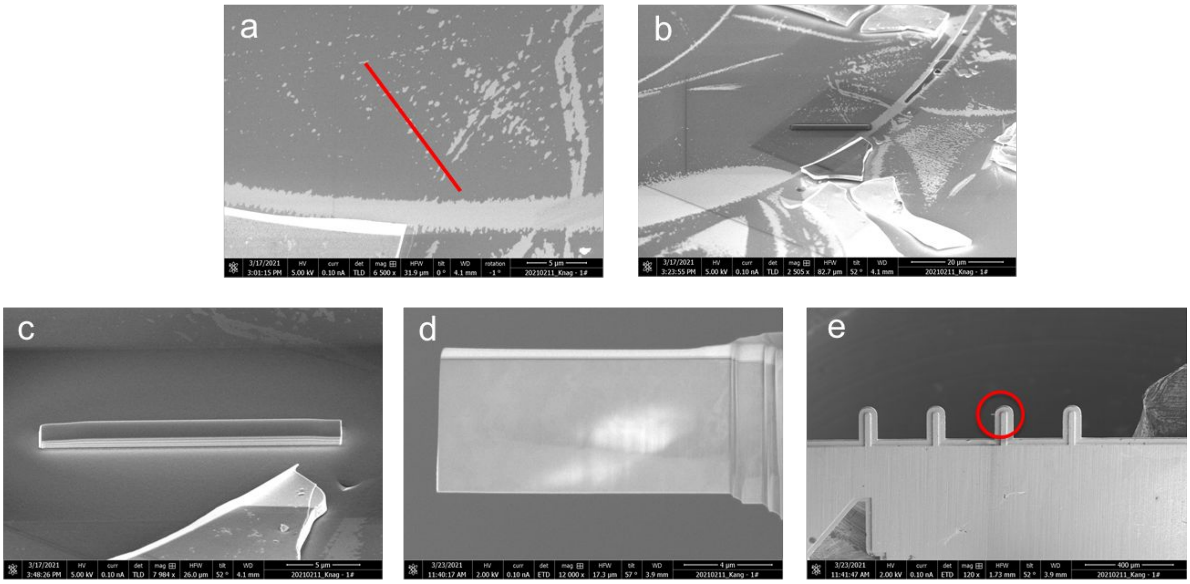

Figure S6. The FIB process to obtain the lamella of surface thin oxidation layer by cutting the surface and exposing the cross section for TEM.

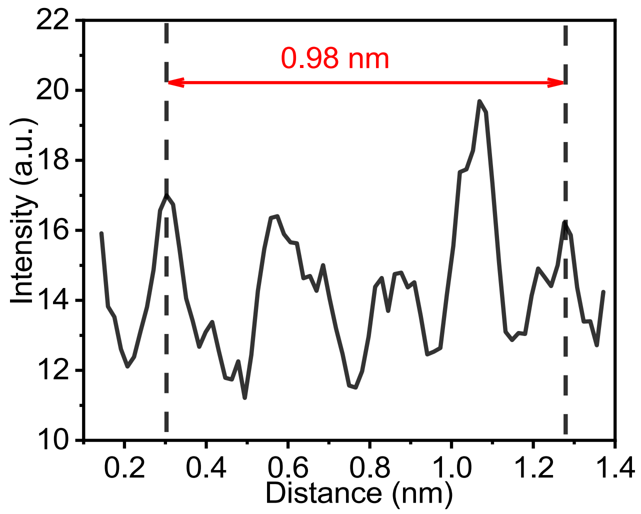

Figure S7. The lattice distance along the red line in the Figure 4c.

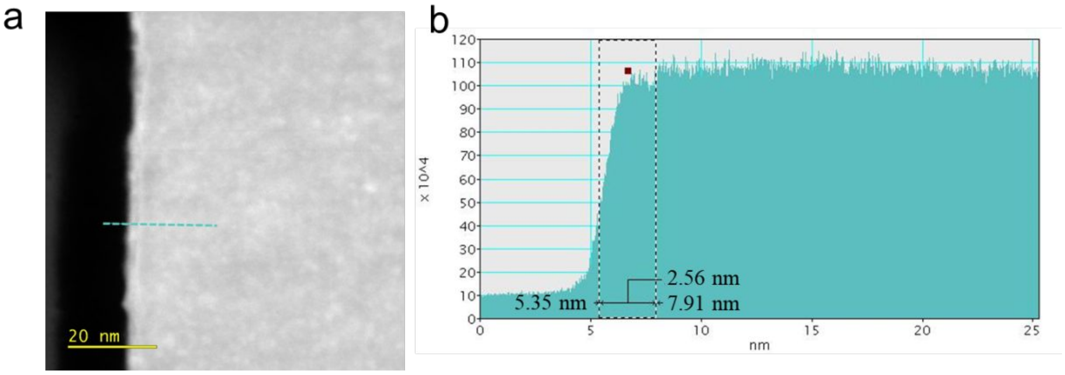

Figure S8. (a) STEM image and (b) the line scanning spectrum of the cross section of CoS_2 after OER.

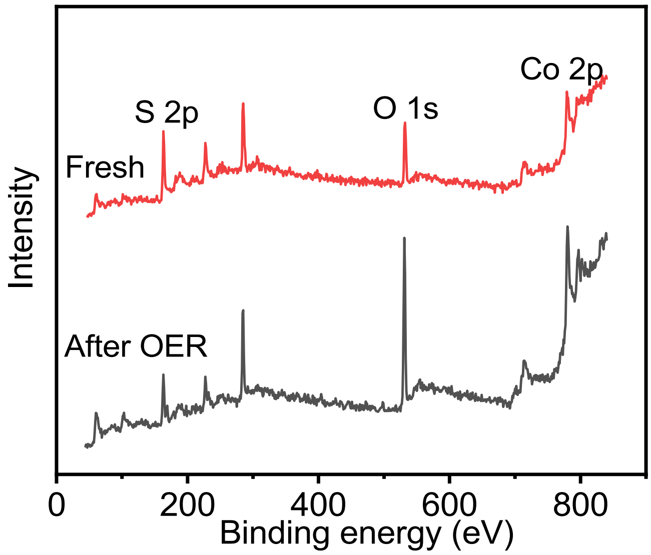

Figure S9. (a) XPS survey of CoS_2 crystal before and after reaction.

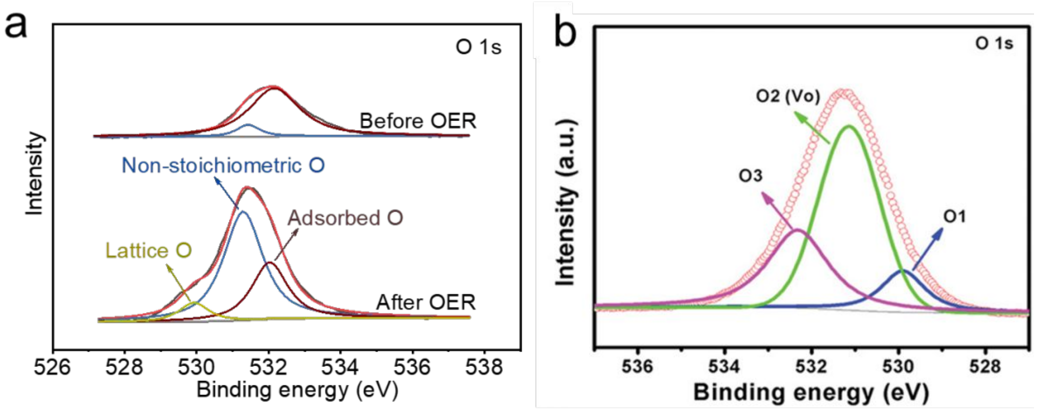

Figure S10. The O 1s XPS spectra in this work (a) and the reference O 1s spectra of Co_3O_4 structure (b). Reprinted with permission from [1]. Copyright [2020] [John Wiley and Sons].

Table S1. Electrochemical impedance spectra fitting parameters in Fig. 2F.

Element	Parameter	Potential		
		1.47 V	1.50 V	1.52 V
R_s	R(Ω)	4.0308	5.9325	6.0648
R_{p1}	R(Ω)	9.2834	7.4568	8.0932
CPE1	Y_0	0.00028833	0.00010184	0.00010911
	N	0.47148	0.60928	0.6105
R_{p2}	R(Ω)	184	89.251	59.684
CPE2	Y_0	0.018031	0.022377	0.018594
	N	0.76186	0.68571	0.66804
	χ^2	0.085	0.053896	0.056102

3. References:

(1) Wang, Q.; Xue, X.; Lei, Y.; Wang, Y.; Feng, Y.; Xiong, X.; Wang, D.; Li, Y. Engineering of Electronic States on Co_3O_4 Ultrathin Nanosheets by Cation Substitution and Anion Vacancies for Oxygen Evolution Reaction. Small 2020, 16, 2001571.
